# Mismatch Repair Protein Expression in Endometrial Intraepithelial Neoplasia and Endometrial Carcinoma: Experience From a Tertiary Care Hospital

**DOI:** 10.7759/cureus.115182

**Published:** 2026-08-25

**Authors:** Ujwala Maheshwari, Ramana Anand, Vrutika Shah

**Affiliations:** 1 Pathology, Mahatma Gandhi Mission Medical College and Hospital, Navi Mumbai, IND

**Keywords:** atypical endometrial hyperplasia, immunohistochemistry, lynch syndrome, mlh1, msh6

## Abstract

Background: Endometrial carcinoma is the most common malignancy of the female genital tract, and endometrial intraepithelial neoplasia (EIN, also termed atypical endometrial hyperplasia) is its recognized precursor lesion. Mismatch repair (MMR) deficiency, identified through loss of MLH1, PMS2, MSH2, and MSH6 expression on immunohistochemistry (IHC), is an important molecular event in endometrial carcinogenesis with diagnostic, prognostic, and hereditary (Lynch syndrome) implications. Comparatively few studies have examined the frequency of MMR loss in EIN. This study evaluated the proportion and patterns of MMR protein loss in EIN and endometrial carcinoma, with descriptive assessment of MMR protein loss according to histopathological grade of endometrial carcinoma.

Methodology: This cross-sectional study was conducted in a tertiary care hospital from August 2024 to December 2025 on 28 endometrial specimens (endometrial biopsies and hysterectomy specimens). These specimens were diagnosed histopathologically as EIN or endometrial carcinoma, collected retrospectively and prospectively using convenience sampling. Formalin-fixed, paraffin-embedded sections were stained with hematoxylin and eosin and evaluated. IHC for MLH1, MSH2, MSH6, and PMS2 was performed on all cases. Complete loss of nuclear staining for any MMR protein with intact internal controls was interpreted as MMR-deficient (dMMR); retained staining for all four proteins was interpreted as MMR-proficient (pMMR). Descriptive assessment of MMR protein loss according to histopathological grade of endometrial carcinoma was also performed.

Results: Of 28 cases, 16 (57.14%) were endometrioid endometrial carcinoma, and 12 (42.86%) were EIN. Patients' ages ranged from 34-68 years; EIN was most common in the sixth decade and carcinoma in the fifth. Among the 16 carcinomas, nine (56.25%) were International Federation of Gynecology and Obstetrics (FIGO) grade 1, six (37.50%) grade 2, and one (6.25%) grade 3. Only one carcinoma (6.25%) was dMMR, showing combined loss of MSH2 and MSH6, and this case was FIGO grade 2. Among the 12 EIN cases, only one (8.33%) was dMMR, showing combined loss of MLH1 and PMS2. All remaining cases in both groups were pMMR.

Conclusions: MMR protein loss was infrequent in this cohort of EIN and endometrial carcinoma, consistent with the broader literature describing MMR deficiency as a relatively uncommon but clinically significant event across the endometrial hyperplasia-carcinoma sequence.

## Introduction

Endometrial carcinoma is the most common malignancy of the female genital tract and a significant cause of morbidity among women worldwide, with an incidence that has risen steadily in association with obesity, metabolic syndrome, diabetes mellitus, and increased life expectancy [1,2]. Most endometrial carcinomas are of endometrioid type and arise from a recognized premalignant precursor, atypical endometrial hyperplasia, now termed endometrial intraepithelial neoplasia (EIN) [1,2]. Bokhman's classical dualistic model divides endometrial carcinoma into estrogen-dependent, endometrioid, type I tumors and estrogen-independent, more aggressive type II tumors; this framework has since been enriched by molecular classification systems, such as the Cancer Genome Atlas (TCGA) and the Proactive Molecular Risk Classifier for Endometrial Cancer (ProMisE), both of which incorporate mismatch repair (MMR) status as a defining molecular parameter [3-5].

The MMR system maintains genomic stability by correcting base-base mismatches and insertion-deletion loops generated during DNA replication. The principal MMR proteins, MLH1, PMS2, MSH2, and MSH6, function as the heterodimeric complexes MutLα (MLH1-PMS2) and MutSα (MSH2-MSH6); loss of MLH1 characteristically produces secondary loss of PMS2, and loss of MSH2 produces secondary loss of MSH6 [6-8]. Defective MMR function results in microsatellite instability (MSI), an accumulation of mutations that drives tumorigenesis, and MMR deficiency has emerged as an important, clinically actionable molecular event identified in a substantial subset of endometrial carcinomas, particularly those of endometrioid histology [9,10].

Loss of MMR protein expression may be sporadic, most commonly due to MLH1 promoter hypermethylation, or may reflect a germline mutation associated with Lynch syndrome, an autosomal dominant hereditary cancer predisposition syndrome in which the lifetime risk of endometrial cancer can reach 40-60% [11-13]. Identification of MMR-deficient (dMMR) tumors is therefore important both for Lynch syndrome screening and because dMMR tumors show increased responsiveness to immune checkpoint inhibitor therapy [12,14]. Immunohistochemistry (IHC) for MLH1, PMS2, MSH2, and MSH6 has become a practical, cost-effective surrogate for MSI testing that additionally identifies the specific protein lost, thereby providing insight into the underlying molecular mechanism, and is now integral to molecular classification and risk stratification in endometrial carcinoma, including the FIGO 2023 staging system [7,15-17].

Although MMR deficiency in endometrial carcinoma has been relatively well characterized, fewer studies have examined its frequency in EIN, the immediate precursor lesion. Evidence suggests that MMR deficiency may occur early in endometrial carcinogenesis, implying that genomic instability can be an early molecular event in tumor progression, and that its detection in EIN may help identify lesions at increased risk of progression and warrant further evaluation for Lynch syndrome [17-19]. This study was undertaken to evaluate the proportion and pattern of MMR protein loss in EIN and endometrial carcinoma using IHC and to descriptively assess the distribution of MMR protein expression according to the histopathological grade of endometrial carcinoma.

## Materials and methods

Study design and setting

This was a combined retrospective and prospective cross-sectional study conducted in the Department of Pathology, MGM Medical College and Hospital, Kamothe, Navi Mumbai, India. The study was conducted from August 2024 to December 2025. The retrospective component comprised archival cases diagnosed between January 2023 and July 2024, from which formalin-fixed, paraffin-embedded blocks and histopathology records of seven cases of EIN and seven cases of endometrial carcinoma were retrieved. The prospective component comprised cases diagnosed between August 2024 and December 2025, during which five additional cases of EIN and nine additional cases of endometrial carcinoma were enrolled using convenience sampling.

Study population

A total of 28 endometrial specimens (endometrial biopsies and hysterectomy specimens) satisfying the inclusion and exclusion criteria were enrolled using a convenience sampling method. Each case represented a unique patient. The participant inclusion criteria included endometrial biopsy or hysterectomy specimens with a histopathological diagnosis of atypical endometrial hyperplasia/EIN or endometrial carcinoma from consenting women above 25 years of age. Patients who were excluded included those with non-atypical endometrial hyperplasia; specimens with diagnoses other than atypical endometrial hyperplasia/EIN or endometrial carcinoma (e.g., endometritis, atrophic endometrium); and specimens that were inadequate, suboptimal, poorly preserved, or showing autolytic change.

Tissue processing and histopathological evaluation

Specimens were fixed in 10% neutral buffered formalin for 6-24 hours, grossed, and processed through an automated tissue processor (dehydration in ascending grades of alcohol, clearing in xylene, and paraffin impregnation at 58-60°C). Paraffin-embedded blocks were sectioned at 3-5 µm, floated on a warm water bath, mounted on glass slides, and stained with hematoxylin and eosin using an automated stainer following standard laboratory protocol. Stained sections were independently examined by two pathologists under light microscopy to confirm histomorphological diagnosis and, for carcinomas, to assign FIGO histological grade based on the proportion of solid, non-glandular growth (grade 1, ≤5% solid; grade 2, 6-50% solid; grade 3, >50% solid), with upgrading by one grade in the presence of marked nuclear atypia.

Immunohistochemistry

Representative formalin-fixed, paraffin-embedded tissue blocks were selected for IHC. Sections of 3-5 µm were cut, mounted on poly-L-lysine-coated slides, and dried at 55-60°C. After deparaffinization and rehydration, heat-induced epitope retrieval was performed using cell conditioning 1 (CC1) on the Ventana Benchmark ULTRA PLUS automated immunostaining platform. Immunohistochemical staining was performed using monoclonal antibodies against the four MMR proteins: MLH1 (clone M1), PMS2 (clone A16-4), MSH2 (clone G219-1129), and MSH6 (clone SP93). All antibodies were Ventana (Roche Diagnostics) ready-to-use (prediluted reagents); therefore, no additional antibody dilution was performed. Endogenous peroxidase activity was blocked, followed by incubation with the primary antibodies and detection using the Optiview DAB detection system (Ventana Medical Systems, Inc., Oro Valley, AZ). Additionally, 3,3'-diaminobenzidine (DAB) was used as the chromogen, with hematoxylin as the counterstain. Nuclear staining in tumor cells was assessed for each of the four MMR proteins. Stromal cells, lymphocytes, and non-neoplastic endometrial glands served as internal positive controls.

Interpretation of MMR IHC

Retained nuclear staining in tumor cells with appropriate internal controls for all four MMR proteins was interpreted as mismatch repair-proficient (pMMR). Complete loss of nuclear staining in tumor cells for any one MMR protein, in the presence of intact internal controls, was interpreted as mismatch repair-deficient (dMMR). All immunohistochemical slides were independently reviewed by two pathologists. In the present study, the staining patterns were conclusive in all cases, and no weak, heterogeneous, or subclonal staining patterns were encountered. No equivocal staining cases requiring additional adjudication were therefore identified. 

Statistical analysis

Data were entered and analyzed using Microsoft Excel (Microsoft® Corp., Redmond, WA). Descriptive statistics were used to summarize the study variables and presented as frequencies and percentages. Given the small sample size and limited number of dMMR cases, no inferential statistical testing was performed, and the relationship between MMR status and FIGO grade was assessed descriptively.

## Results

Among EIN cases, the age distribution was as follows: 30-40 years, one case (8.33%); 41-50 years, four cases (33.33%); 51-60 years, six cases (50.00%); and 61-70 years, one case (8.33%). Most EIN cases therefore occurred in the sixth decade of life (Table 1).

**Table 1:** Age-wise distribution of endometrial intraepithelial neoplasia (EIN) (n=12)

Age group (years)	Number of EIN cases	Percentage
30-40	1	8.33%
41-50	4	33.33%
51-60	6	50.00%
61-70	1	8.33%

Among endometrial carcinoma cases, the age distribution was: 30-40 years, four cases (25.00%); 41-50 years, six cases (37.50%); 51-60 years, four cases (25.00%); and 61-70 years, two cases (12.50%). Most carcinoma cases therefore occurred in the fifth decade of life (Table 2).

**Table 2:** Age-wise distribution of endometrial carcinoma (n=16)

Age group (years)	Number of carcinoma cases	Percentage
30-40	4	25.00%
41-50	6	37.50%
51-60	4	25.00%
61-70	2	12.50%

All 16 endometrial carcinoma cases were of endometrioid histological type, and FIGO grading was available for all cases. Nine cases (56.25%) were grade 1, six (37.50%) were grade 2, and one (6.25%) was grade 3, indicating a predominance of low-grade, well-differentiated tumors in this cohort. The single carcinoma case that showed combined loss of MSH2 and MSH6 belonged to FIGO grade 2 (Table 3).

**Table 3:** Distribution of endometrial carcinomas by FIGO histological grade (n=16) FIGO: International Federation of Gynecology and Obstetrics

FIGO grade	Number of cases	Percentage
Grade 1	9	56.25%
Grade 2	6	37.50%
Grade 3	1	6.25%

Among the 16 FIGO-graded carcinomas, nine (56.25%) pMMR cases were grade 1, five (31.25%) were grade 2, and one (6.25%) was grade 3. The one (6.25%) dMMR case was grade 2 (Table 4).

**Table 4:** MMR protein expression according to FIGO histopathological grade (n=16) FIGO: International Federation of Gynecology and Obstetrics; IHC: immunohistochemistry; MMR: mismatch repair

MMR IHC expression	Grade 1	Grade 2	Grade 3
MMR proficient (pMMR)	9	5	1
MMR deficient (dMMR)	0	1	0

Of the 12 EIN cases, 11 (91.67%) were pMMR, and one (8.33%) was dMMR. The single dMMR EIN case showed concurrent loss of MLH1 and PMS2, a pattern more frequently associated with sporadic MLH1 promoter hypermethylation. No cases of isolated PMS2 or isolated MSH6 loss were identified (Table 5).

**Table 5:** MMR status and pattern of MMR protein loss in endometrial intraepithelial neoplasia (EIN) (n=12) MMR: mismatch repair

MMR status	Number of cases	Percentage	MMR gene(s) lost
MMR proficient (pMMR)	11	91.67%	-
MMR deficient (dMMR)	1	8.33%	MLH1 and PMS2

Of the 16 endometrial carcinoma cases, 15 (93.75%) were pMMR, and one (6.25%) was dMMR. This single dMMR carcinoma showed concurrent loss of MSH2 and MSH6, a pattern more frequently associated with Lynch syndrome. No cases of isolated PMS2 or isolated MSH6 loss were identified (Table 6).

**Table 6:** MMR status and pattern of MMR protein loss in endometrial carcinoma (n=16) MMR: mismatch repair

MMR status	Number of cases	Percentage	MMR gene(s) lost
MMR proficient (pMMR)	15	93.75%	-
MMR deficient (dMMR)	1	6.25%	MSH2 and MSH6

**Figure 1:**
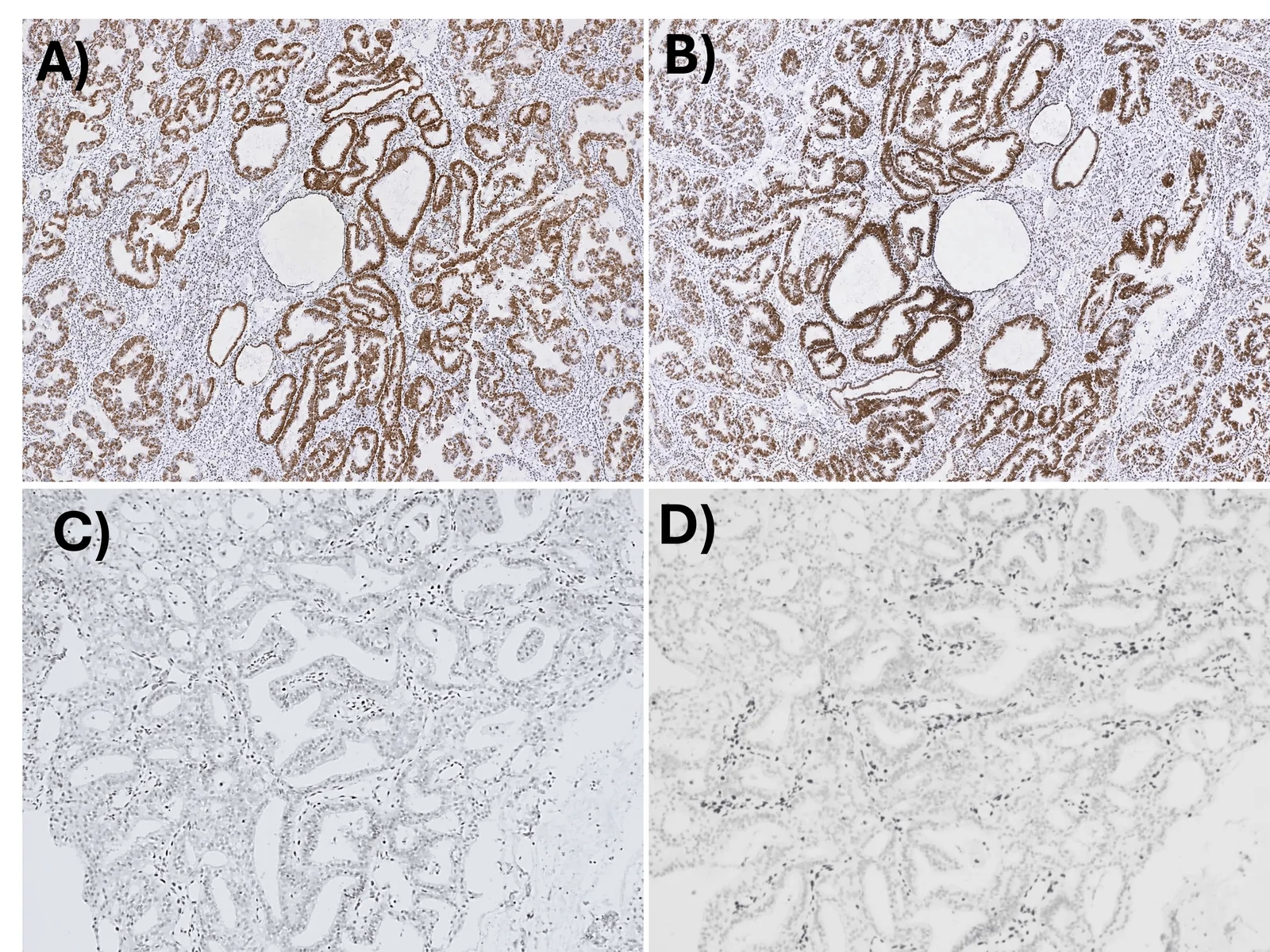
Histopathology images A) Retained MSH2 nuclear expression in EIN of a 56-year-old woman. B) Retained MSH6 nuclear expression in EIN in the same case. C) Loss of MLH1 nuclear expression, with intact internal controls, in a 60-year-old woman with EIN. D) Loss of PMS2 nuclear expression, with intact internal controls, in the same case.

Additionally supporting the results, Figures 1A-1B show retained MSH2 and MSH6 expression, respectively, in a 56-year-old female with endometrial EIN. Figures 1C-1D show loss of MLH1 and PMS2 expression, respectively, with intact internal controls in a 60-year-old female with endometrial EIN. Note that, in both Figures 1C-1D, the endometrial glands show no IHC staining; however, the stroma shows positive internal controls.

## Discussion

MMR proteins maintain genomic stability by correcting base-base mismatches and insertion-deletion loops arising during DNA replication. Within the endometrial hyperplasia-carcinoma sequence, EIN represents the recognized precursor of endometrioid carcinoma and shares molecular alterations with the invasive tumors it precedes. Prior work has suggested that MMR protein loss can occur at this premalignant stage, supporting the concept that genomic instability may be an early event in endometrial tumorigenesis, although the reported frequency of MMR loss in EIN varies across studies, indicating that not all premalignant lesions progress through the MSI pathway [17-19].

In the present study, the mean age of women with EIN was higher than that reported by Nees et al. (45.2 years) and closer to, though somewhat older than, that reported by Han et al. (49 years) [19,20]. Similarly, the mean age of women with endometrial carcinoma in this cohort was younger than that reported by Agarwal et al. (54 years) and considerably younger than that reported by Sorosky (61 years) [21,22]. The comparatively younger age distribution observed here, together with similar trends reported from other Indian cohorts, suggests that endometrial carcinoma may present at a younger age in Indian populations than in some Western series, reinforcing the need for a high index of suspicion for endometrial pathology in relatively young women presenting with abnormal uterine bleeding.

The FIGO grade distribution in this study, with a predominance of grade 1 tumors (56.25%), followed by grade 2 (37.50%) and grade 3 (6.25%), was broadly comparable to that reported by Grevenkamp et al. (41.3% grade 1, 51.7% grade 2, and 6.9% grade 3) and by Agarwal et al. (44% grade 1, 39.5% grade 2, and 16.5% grade 3), reinforcing the well-established observation that most endometrial carcinomas are low-grade endometrioid neoplasms with favorable biological behavior [21,23].

MMR deficiency was uncommon in this cohort, identified in only 6.25% of endometrial carcinomas and 8.33% of EIN cases. This proportion in carcinoma is lower than the 22-33% frequently reported in larger published series, a discrepancy most plausibly attributable to the small sample size of this study, in which a single positive case exerts a disproportionate effect on the overall percentage [9]. The proportion identified in EIN (8.33%) was comparable to, albeit somewhat higher than, the 4.5% reported by Nees et al., again reflecting the influence of a single case in a small cohort [19]. Both findings nevertheless support the broader literature indicating that MMR deficiency, while an early and biologically important event in a subset of lesions, affects only a minority of premalignant endometrial lesions [17-19].

The pattern of MMR protein loss observed differed between the two lesion groups. The dMMR EIN case showed combined MLH1/PMS2 loss, a pattern commonly associated with sporadic MLH1 promoter hypermethylation, consistent with the predominant mechanism reported in comparable series [11]. However, molecular confirmation was not performed in this study. In contrast, the dMMR carcinoma showed combined MSH2/MSH6 loss, a pattern more strongly associated with germline mutation and Lynch syndrome than with epigenetic silencing [11-13], although germline testing was not performed in this study. This finding, even though based on a single case, is clinically noteworthy: MSH2/MSH6 loss in endometrial carcinoma should prompt further clinical evaluation, including assessment of family history and referral for genetic counselling and testing, given the substantially elevated lifetime risk of endometrial and colorectal malignancy associated with Lynch syndrome [11-13].

With respect to grade, the single dMMR carcinoma in this study was FIGO grade 2, in keeping with published observations that MMR-deficient tumors may occur more frequently among low- and intermediate-grade endometrial carcinomas than among high-grade tumors [9]. The small number of dMMR cases identified precluded meaningful assessment of the association between MMR status and FIGO grade in this cohort, and this observation should therefore be interpreted as hypothesis-generating rather than confirmatory.

The functional interdependence of the MMR heterodimers, MLH1 with PMS2 and MSH2 with MSH6, has direct implications for immunohistochemical interpretation: loss of MLH1 is typically accompanied by secondary loss of PMS2, whereas isolated PMS2 loss suggests a primary PMS2 mutation, and concurrent MSH2/MSH6 loss suggests an MSH2 mutation, whereas isolated MSH6 loss points to a primary MSH6 defect [8,12]. Recognition of these patterns, together with correlation with clinical and family history, is essential for appropriately triaging patients for confirmatory MLH1 methylation analysis or germline genetic testing [12,13,24].

Beyond its diagnostic and hereditary-screening value, MMR status carries prognostic and therapeutic significance. MMR-deficient tumors demonstrate a higher mutational burden and increased tumor-infiltrating lymphocytes, which underlie their enhanced responsiveness to immune checkpoint inhibitor therapy targeting the PD-1/PD-L1 pathway, and MMR status is now formally incorporated into the FIGO 2023 molecular classification of endometrial carcinoma alongside POLE-mutated, no specific molecular profile (NSMP), and p53-abnormal categories [10,14,15]. This highlights the potential clinical utility of MMR immunohistochemistry in the pathological evaluation of EIN and endometrial carcinoma.

Limitations

This study is limited by its small sample size, single-institution design, and the resultant inability to perform meaningful statistical assessment between MMR status and clinicopathological parameters. Molecular confirmation of MLH1 promoter hypermethylation and germline genetic testing were not performed, and the observed patterns of MMR loss, while consistent with expected mechanisms, remain inferential. Larger multi-institutional studies incorporating confirmatory molecular testing are needed to validate these observations.

## Conclusions

In this cohort, MMR protein loss was infrequent in both EIN and endometrial carcinoma, with most cases of both lesion types occurring in peri-menopausal women and most carcinomas being of endometrioid histology and low FIGO grade. The single dMMR EIN case showing MLH1/PMS2 loss is consistent with the possibility that MMR deficiency may occur as an early molecular event in endometrial tumorigenesis, while the MSH2/MSH6 loss identified in the single dMMR carcinoma warrants consideration of Lynch syndrome and highlights the clinical relevance of MMR immunohistochemistry. Immunohistochemistry for MLH1, PMS2, MSH2, and MSH6 remains a simple and cost-effective method for identifying MMR-deficient lesions, with diagnostic, prognostic, therapeutic, and hereditary-screening implications. Despite the limitations of a small sample size, these findings highlight the potential utility of MMR immunohistochemistry in the evaluation of EIN and endometrial carcinoma and emphasize the need for larger studies incorporating molecular confirmation.
